# A Semi-recumbent Eccentric Cycle Ergometer Instrumented to Isolate Lower Limb Muscle Contractions to the Appropriate Phase of the Pedal Cycle

**DOI:** 10.3389/fphys.2021.756805

**Published:** 2021-11-29

**Authors:** Joel A. Walsh, Darryl J. McAndrew, Douglas J. Henness, Jonathan Shemmell, Dominic Cuicuri, Paul J. Stapley

**Affiliations:** ^1^Neural Control of Movement Laboratory, School of Medicine, Faculty of Science, Medicine and Health, University of Wollongong, Wollongong, NSW, Australia; ^2^Electrical Workshop, Faculty of Engineering and Information Sciences, University of Wollongong, Wollongong, NSW, Australia; ^3^Neuromotor Adaptation Laboratory, School of Medicine, Faculty of Science, Medicine and Health, University of Wollongong, Wollongong, NSW, Australia

**Keywords:** eccentric muscle contraction, cycle ergometer, servo-drive, electromyography, motion control

## Abstract

Eccentric (ECC) cycling is used in rehabilitation and sports conditioning settings. We present the construction and mode of operation of a custom-built semi-recumbent ECC cycle designed to limit the production of lower limb muscle activity to the phase of the pedal cycle known to produce ECC contractions. A commercially available semi-recumbent frame and seat (Monarch, 837E Semi-recumbent Bike, Sweden) were used to assemble the ergometer. An electrical drive train system was constructed using individual direct drive servo motors. To avoid active muscle activation occurring during the non-ECC pedaling phase of cycling, a “trip” mechanism was integrated into the drivetrain system using a servo-driven regenerative braking mechanism based on the monitoring of the voltage produced over and above a predetermined threshold produced by the motors. The servo drive internal (DC bus) voltage is recorded and internally monitored during opposing (OPP) and non-opposing (N-OPP) phases of the pedal cycle. To demonstrate that the cycle functions as desired and stops or “trips” when it is supposed to, we present average (of 5 trials) muscle activation patterns of the principal lower limb muscles for regular ECC pedal cycles in comparison with one pedal cycle during which the muscles activated outside the desired phase of the cycle for a sample participant. This semi-recumbent ECC cycle ergometer has the capacity to limit the occurrence of muscle contraction only to the ECC phase of cycling. It can be used to target that mode of muscle contraction more precisely in rehabilitation or training studies.

## Introduction

Semi-recumbent eccentric (ECC) cycling is frequently used in rehabilitation primarily due to the significantly reduced cardiorespiratory demand at a given workload, compared to traditional concentric (CON) cycling (Dufour et al., 2004; Hoppeler, 2016). Previous ECC cycling studies have shown significant neuromuscular and musculoskeletal improvements among clinical populations (Dibble et al., 2006, 2009; Gerber et al., 2007; Elmer et al., 2010; Chasland et al., 2017; MacMillan et al., 2017; Lewis et al., 2018). Additional studies have also investigated whether ECC cycling-induced lower limb strength adaptations translate into improved exercise performance including cycling power outputs and squat and countermovement jump height (Gross et al., 2010; Leong et al., 2014; Paulsen et al., 2019).

Eccentric contractions involve the active lengthening of a muscle when an applied force exceeds the force produced by the muscle (Lindstedt et al., 2001; Vogt and Hoppeler, 2014). During ECC cycling (either upright or semi-recumbent), participants perform repetitive ECC muscle contractions by repeatedly applying resistive force against backward-rotating motor-driven pedals (Green et al., 2018; Barreto et al., 2021). Primarily, the knee extensor muscles (i.e., quadriceps) act as a braking force by absorbing the load from the motor-driven pedals of an ECC cycle ergometer (Elmer et al., 2010; Peñailillo et al., 2017). More specifically, ECC contraction of the knee extensors occurs within the “pushing” or “ECC extension” phase of ECC cycling (Elmer et al., 2010; Peñailillo et al., 2015), as the motor-driven pedals move from bottom dead center (BDC) to top dead center (TDC; Green et al., 2018). Therefore, during ECC cycling, ECC muscle contractions can only occur when the backward-rotating pedal becomes opposable [as the backward pedal moves from slightly beyond BDC to TDC; opposing (OPP) phase], thus allowing the rider to apply an OPP, braking force. Alternatively, muscle contractions that occur when the pedal is *not* an OPP force (from TDC to slightly beyond BDC; non-opposing (N-OPP) phase cannot be considered ECC, as the knee extensors would be shortening in length (i.e., CON contraction).

It has been suggested that ECC cycle ergometers allow ECC muscle contractions to be completed with limited CON contractions (Green et al., 2018). However, where ECC cycle ergometers operate using a motorized chain drive system consisting of a dual-sprocket and chain configuration, similar to a regular bicycle (Elmer and Martin, 2013; Lewis et al., 2018), this cannot be guaranteed. A limitation of a chain drive system is that power transferred from the pedals to the chain is not retained by the system and lost, for example, as frictional heat (Spicer et al., 1999). This may be considered non-critical during CON cycling, where power transfer is generated wholly by CON muscle contractions. During ECC cycling however, to ensure that force is not applied during the N-OPP phase of an ECC pedal cycle, such a transfer of power into the system can be used to establish a threshold above which power to the motor-driven pedals is cut. Such a system (described here) would ensure that muscular contractions are concentrated to the OPP phase of an ECC pedal cycle only, and any conclusions about the clinical or training implications of ECC work assume that the activity of the major contributing muscle groups is in fact, ECC in nature.

Therefore, the purpose of this special communication is to outline the design and control mechanisms of a novel semi-recumbent ECC cycle ergometer that minimizes N-OPP pedal phase activity by using the power transferred through the pedals to the drive system as a regenerative “trip” mechanism to arrest the motor-driven pedals. We describe the construction from a commercially available recumbent cycle, the basic circuitry involved in controlling the “trip” mechanism, and illustrate electromyography (EMG) of major muscle groups during pedaling that would activate such a mechanism.

## Materials and Methods

### Conversion From the Original Cycle Ergometer

**Figure 1:**
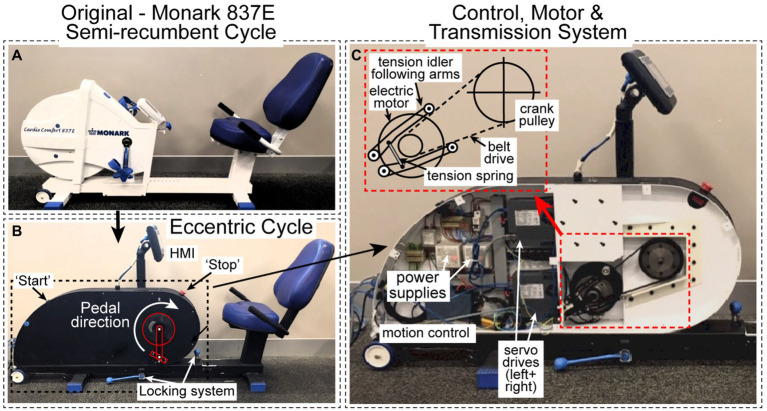
**(A)** The original Monark 837E recumbent ergometer. **(B)** The modified semi-recumbent ECC cycle ergometer with the replacement chassis was installed on to the original frame. Start and stop buttons, locking system, HMI display screen, and pedal direction are all shown. **(C)** The mechanical contents housed in the new chassis (motion controllers, left and right servo drives). The insert shows the independent closed loop system, tension idlers, electric motor, and belt drive attached to the crank pulley (left side only, replicated on right side – not shown).

A commercially available semi-recumbent bike frame (Monark, 837E Semi-recumbent Bike, Sweden; Figure 1A) was used as the basis for construction of the semi-recumbent ECC cycle ergometer (Figure 1B). The flywheel, drivetrain (including cranks), and display unit were removed from the original ergometer, and the frame, seat, adjustable back support, and supporting handles were preserved. The chassis was modified so that it can be moved along the frame and locked in place at 50-mm intervals to accommodate subject height and leg length (adjustable difference between pedals and seat is 490–890mm). The mechanical contents, including servo motors, servo drive controllers and motion control coordinators are housed in the new chassis (Figure 1C).

### Motor and Transmission System

**Figure 2:**
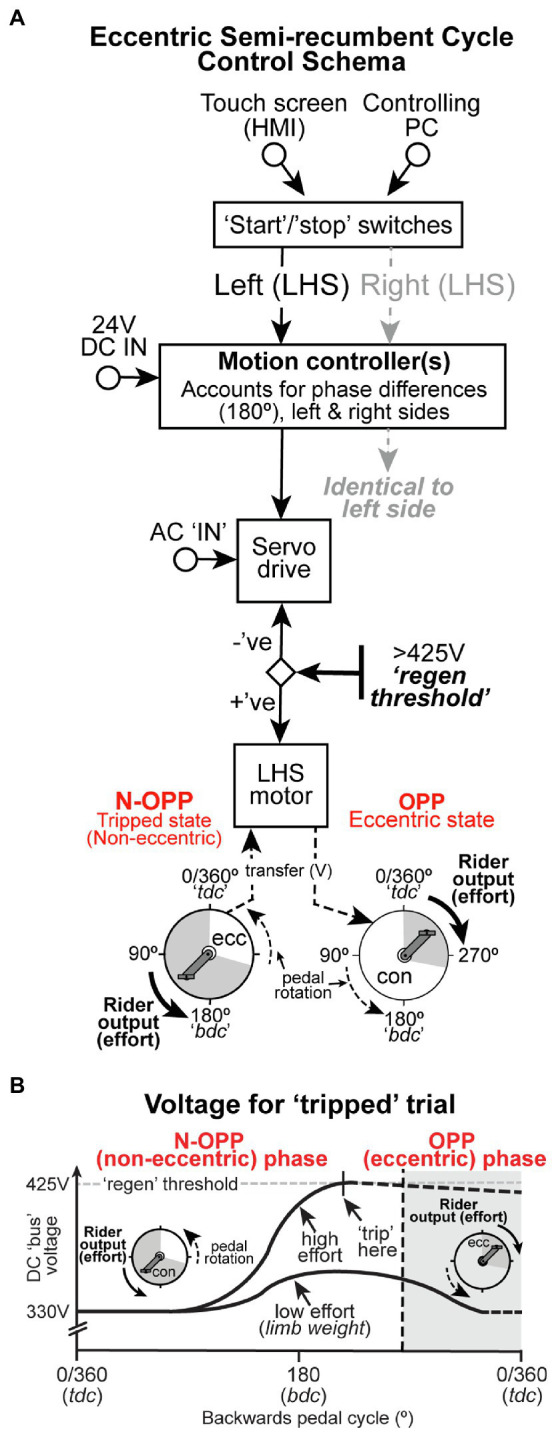
**(A)** The cycle control schema. For a detailed explanation, see “Materials and Methods.” For both left and right sides, a non-opposed (N-OPP) and opposed (OPP) state of the ECC pedal cycle is set whereby the voltage produced by the motor is summed with that exerted back to the system and compared to a predetermined threshold. When rider output is added to the motor output voltage (N-OPP), the system trips or stops. When rider output is opposite to pedal rotation (OPP), the system does not trip. **(B)** Theoretical illustration of voltage changes associated with a tripped pedal cycle. For a detailed explanation, see “Materials and Methods.” High effort represents a situation in which the rider exerts muscle force to the pedal during the N-OPP (non-eccentric) phase. Low effort represents the change in voltage induced by the weight of the limb which would not trip the cycle.

Two direct drive servo motors (Hans type FI3-015-S-A-1, Motion Technologies, Caringbah, NSW, Australia), of 300rpm and 50N·m peak torque capacity, 600W peak power output, control left-hand side (LHS) and right-hand side (RHS) drivetrain systems so that the pedals can be driven separately (Figures 1C, 2). Each servo motor has a small rotor size (Ø 25mm) and low inertia (0.0049kgm^2^). This combination enables fast and accurate acceleration/deceleration and almost instantaneous stopping. Each motor was factory calibrated with a reported repeatability and positioning accuracy of ±2° and±30 arc·s^−1^, respectively. Timing belts (Gates 825-5M-15 PowerGrip HTD Belt, Denver, CO, United States) form independent, closed loop LHS and RHS drivetrain systems between the respective left and right-side crank pulley and servo motors (Figure 1C), with a gearbox ratio of 4:1. Tension idlers (Figure 1C, insert) were fitted to each system to accommodate belt tension allowing a smooth rotation of the drivetrain, as well as achieving the desired belt routing during cycling. Each independent crank pulley axle was fitted with a 175mm length crank and flat-surface steel pedal. Power is supplied to the ergometer through an Australian/New Zealand AC power plug (AS/NZS 3112 Type I, 230V, 50Hz) and operated through the start/emergency stop circuit (Figures 1B, 2) of an internal electrical relay.

Laptop-based software (Motion Perfect v4.3, Trio Motion Technology, Gloucestershire, United Kingdom) is used to configure and program the motion control coordinator *via* a standard EtherCAT connection. The cycle ergometer can be manually operated as a “standalone” system using the color touch screen interface (TRIO UNIPLAY HMI 7″, Trio motion technology, Gloucestershire, United Kingdom). Electrical command signaling from the two-axis motion control coordinator (TRIO MC4N EtherCAT Controller, Trio motion technology, Gloucestershire, United Kingdom) is relayed to independent 120/240VDC – 1/3 phase, 2000W continuous power output servo drives (AKD, Kollmorgen, Radford, VA, United States) that govern the LHS and RHS drivetrain systems. Each servo drive provides accurate drive direction, speed, acceleration, torque, and power data in real time at a maximal sampling rate of 8Khz resolution (×128) or 1,515,520 positions/revolution. The motion control coordinator has eight in-built 24V inputs and eight bi-directional I/O channels. A watchdog circuit is embedded into the transmission system to safeguard, detect, and recover the motion control coordinators from malfunctions. A hard-wired emergency stop circuit is housed between the servo drives and motors of each side and when pressed, immediately halts power to the entire transmission system. The emergency stop can easily be accessed by the rider or a supervising researcher. To further safeguard the rider from any sudden acceleration, the first 20s of all semi-recumbent ECC cycling exercise is used to gradually accelerate to the pre-selected motor speed (rpm).

### Manual Control System, Start-Up, and Data Export

The cycle ergometer is operated using a color touch screen interface (TRIO UNIPLAY HMI 7″, Trio motion technology, Gloucestershire, United Kingdom) and commanded by a Windows operating system (Microsoft Corporation, Redmond, WA, United States). Screen interfaces are created and configured on a laptop PC using the Motion Perfect v4.3 software. Interfaces are then stored on to the motion control coordinator and displayed on the HMI touch screen while running.

After starting up the semi-recumbent ECC cycle ergometer, HMI interfaces are used to perform a system initialization sequence (i.e., initializing the EtherCAT connection and homing each motor position), set motor speed (i.e., isokinetic – cadence/rpm), and zero the left-hand side (LHS) and right-hand side (RHS) motor positions. A “home” HMI interface is used to operate the LHS and RHS motors, to display real-time data, and to save and export data files. Ride data containing torque (N·m), power output (W), position (°), angular velocity (rad/s), cadence (rpm), and time for each motor can be saved to and stored on a 4 Gb FAT32 format SD card (element14, Transcend Information Inc., Santa Clara, CA, United States). Saved data are exported in a text file format.

### Regenerative Braking–The “Trip” Mechanism

The servo motors are controlled by their respective servo drives and are electrically powered with the capacity to act as electric generators that recover kinetic energy to slow or provide braking force to a moving part. This is commonly referred to as regenerative braking. In the current ergometer, kinetic energy that is generated through power transferred to the drive system from muscular activity is retained as increased voltage on the internal DC bus and used in comparison with a manufacturer-determined threshold of >425V (see Figure 2A). If the threshold is exceeded, the servo motors are deactivated.

During ECC pedaling, force is typically applied when the backward moving pedal (Gross et al., 2010) provides an opposable force. To define where the pedal is opposable within an ECC pedal revolution (see Figure 2A), we have considered an ECC pedal revolution as: 1) a complete 360° revolution of the left or right pedal as rotating from TDC (0°), beyond BDC (180°) and returning to TDC (Hug et al., 2008); 2) where crank angle increases as the pedal/crank moves backward; and 3) the opposable phase (OPP phase, i.e., where the pedal is an opposable force) occurring at a crank angle range from 260–360° (see Figures 2A,B “OPP – ECC state”). The OPP phase crank angle range (i.e., 260–360°) corresponds to the absolute knee angle range (20–90°) where ECC torque and muscle recruitment activity, in vastus lateralis, are greatest (peak at 70°) during semi-recumbent ECC cycling (Peñailillo et al., 2015). Beyond this range, any applied force would occur in the N-OPP phase and therefore assist the direction of the motor-driven pedals. Since the servo drive aims to maintain the pedal speed at the desired value, this results in regeneration (“regen”). This captured energy (from muscle activity) which attempts to increase pedal speed results in an increase of the DC bus voltage described above (see N-OPP, left side Figures 2A,B). This would theoretically involve non-ECC activity of lower limb muscles.

The aim of this cycle was to use the power transferred to the electrical system during the OPP phase and compare it to the factory set threshold of 425V. If the voltage exceeds that threshold during the N-OPP phase (see theoretical “Tripped state,” bottom Figures 2A,B), the system will stop the cycle (“trip”) and ECC cycling will cease until the ergometer is reset to its start position. We have therefore incorporated “regenerative braking” into the cycle to prevent it being driven during the N-OPP phase when rider output (effort) adds to the voltage threshold, thus reducing or eliminating CON contractions of the lower limb muscles. Alternatively, kinetic energy (excess voltage) generated during the OPP phase of the ECC pedal cycle is directed to resistors that temporarily store that voltage, rather than tripping the cycle (Figure 2). Alternatively, muscle activity applied between 260 and 360° during the OPP phase produces a retardation force to the pedaling direction. The servo drive applies effort against the muscle action, and the resultant motoring action (Figures 2A, B) does not invoke regeneration, thus the internal DV bus voltage remains at 330VDC (rectified value of 240VAC supply). It should be noted that the collective weight of the rider’s lower limb is not sufficient to breach the 425V threshold at any phase of the pedal cycle (see “low effort” representation in Figure 2B).

### Illustrative Participant Riding the Cycle

To illustrate typical muscular contractions of the lower limb muscles and activity associated with a “trip” of the system, representative data were obtained from a healthy participant (aged 33years, mass=98.0kg, height=183.3cm, BMI=29.26kg/m^2^). The participant completed a Sports Medicine Australia questionnaire to determine exercise readiness. Procedures were approved by the University’s Human Research Ethics Committee and carried out in accordance with the Declaration of Helsinki. Written informed consent was obtained from the participant, prior to data collection.

### Surface EMG

Muscle activation patterns were recorded using surface EMG from seven muscles [biceps femoris (long head), BF; rectus femoris, RF; vastus lateralis, VL; vastus medialis, VM; soleus, S; medial gastrocnemius, MG; and tibialis anterior, TA] of the dominant leg (Zych et al., 2018), based on their primary involvement in semi-recumbent cycling (Hakansson and Hull, 2005). Prior to electrode placement, skin sites were prepared by shaving, mildly abrading and cleansing with isopropyl alcohol to improve electrode-skin contact. A single 10mm diameter Ag/AgCl bipolar rectangular bar electrode (Bagnoli^™^, Delsys Incorporated, Natick, MA, United States) was positioned over the muscle belly and parallel to the direction of the respective muscle fibers, by the same researcher, in accordance with the recommendations by Surface EMG for Non-Invasive Assessment of Muscles (SENIAM guidelines). The reference electrode was fixed over the right clavicle. EMG signals were sampled at 2,000Hz, gain amplified (1000×), digitized using a 16-bit analogue-to-digital converter (Power1401, Cambridge Electronic Design, Cambridge, United Kingdom) and exported for offline analysis. Offline analysis was performed using Spike software version 6.02 (Cambridge Electronic Design, Cambridge, United Kingdom). Processing of EMG data involved full-wave rectification, DC removal, and band-pass filtering between 10 and 500Hz using a 4^th^ order low-pass Butterworth filter (high pass 0.5dB and low pass 20dB) in accordance with procedures (Chapman et al., 2008; Hug et al., 2008) outlined by the International Society of EMG and Kinesiology (Merletti and Di Torino, 1999). Crank positions were determined using trigger pulses at 0/360° and 180° based on the aforementioned considerations of an ECC pedal revolution. Processed EMG signals were segmented using this method in order to define muscle activation patterns over a single pedal revolution.

### Semi-recumbent ECC Cycling Workload

The participant performed 15min of isokinetic semi-recumbent ECC cycling at an individually prescribed power output (328 Watts, fixed cadence of 60rpm). The prescribed power output was a summation of 10% of the participant’s peak power output value (3,013 Watts), recorded during a peak test (PETP) specific to the semi-recumbent position (Walsh et al., 2021), and the zero-offset value (27 Watts). In line with industry procedure, zero offsetting defines a zero “0” baseline value for recording torque and power output values. For this ergometer, zero offsetting accounts for the torque exerted on the motor by the additional combined weight of the cranks and pedals. Zero offsetting is conducted over a 60s period prior to ECC cycling, in an unloaded (no weight) state. The average power output recorded during the zero-offset procedure is added to the rider’s peak PETP value, as the zero-offset value was not considered during the factory calibration of the servo motors.

## Results

### Typical Electromyographic Activity Recorded During Pedaling the Ergometer

**Figure 3:**
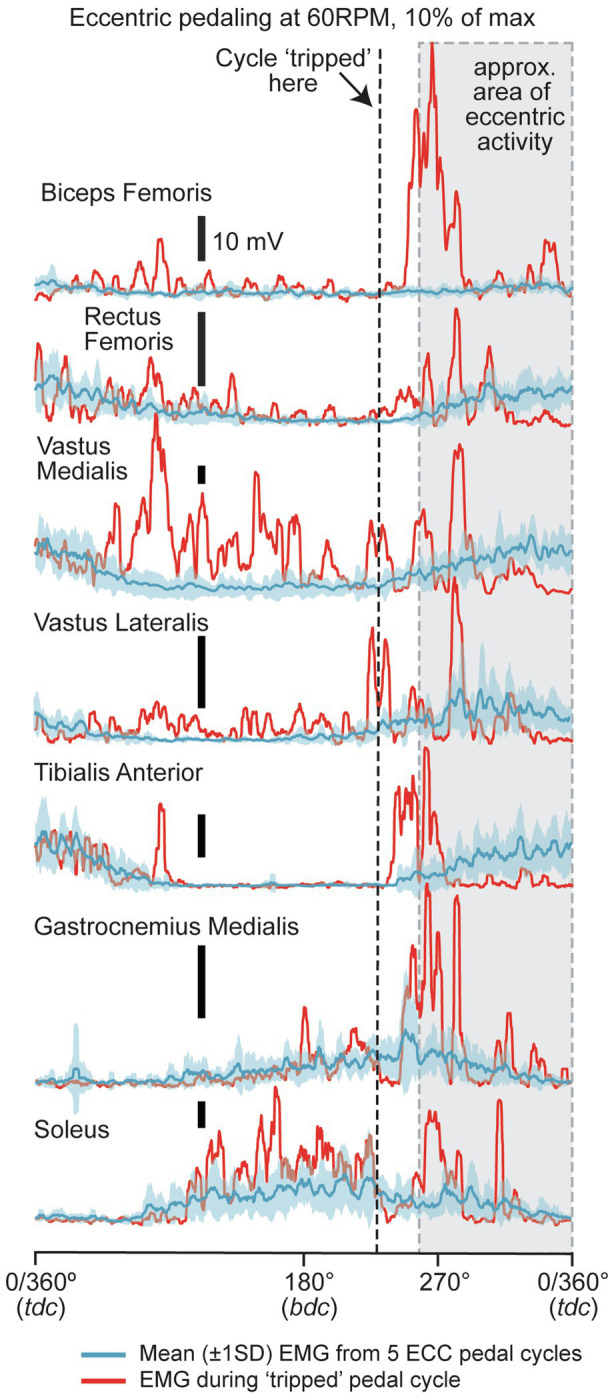
Mean (±1SD) EMG traces for a single participant for regular ECC cycling (mean= blue, ±1SD=light blue envelope) for BF, RF, VM, VL, TA, GASM, and SOL muscles. The mean (±1SD) of five ECC pedal cycles are shown. The red traces for each muscle represent one single pedal cycle that “tripped” the cycle. In all pedal cycles, ECC cycling was performed at 60rpm and at 332W (prescribed workload was 328W. The vertical dashed line represents the time that the cycle was “tripped” (red traces). The grey area represents the documented range of the ECC pedal cycle that ECC muscle activity is produced (Peñailillo et al., 2015).

The participant maintained an average ECC power output of 332 Watts, at 60rpm, for the 15min of cycling. The averaged ECC power output differed by 1.24% from the prescribed power output of 328Watts. Muscle activation patterns for the representative participant are presented in Figure 3 (average of five trials). Average (±1SD) non-trip EMG waveforms for five pedal cycles are displayed in Figure 3. Rectus femoris, VM, VL, and TA (and to a lesser extent GASM) showed increases in activity between approximately 260° and 340–360° in line with known ECC muscle activation patterns (Peñailillo et al., 2015; Green et al., 2018). It should be noted that activity was also recorded in the SOL muscle outside the area of ECC activity (OPP phase), probably to control pedal position around BDC.

### Illustrative Electromyographic Activity of a “Trip” Trial

The participant “tripped” the ergometer once, at 13:47min (808th s) during 15min of ECC cycling. A typical trial during which the cycle “tripped” or stopped is shown on Figure 3 (red traces). In this pedal cycle, the “trip” occurred at around 225°. The greatest muscle activity during the phase between 0 to 225°, which did not correspond to the regular (averaged) activity when the cycle did not trip, could be seen in the VM, VL, and to a lesser extent, the BF, RF, and TA. Therefore, it is highly likely that this muscle activity produced over and above the regular activity during a preferred “ECC state” and resulted in power being transferred to the drive system and exceeding the regenerative threshold, triggering the DC link, and stopping the motors. After the cycle “tripped” (vertical dashed line for the red traces in Figure 3), there was general co-contraction of all muscles to control the limb as it was suspended due to no support being provided by the pedal.

## Discussion

We have outlined the construction of a novel semi-recumbent ECC cycle ergometer, specifically designed to limit semi-recumbent ECC cycling to only ECC muscle contractions during a phase where participants opposed the direction of the pedals. To our knowledge, this is the first ECC cycle ergometer that has been purposely built with an integrated mechanism designed to minimize the potential for non-ECC muscle activations occurring during the non-OPP phase of semi-recumbent ECC cycling.

As previously mentioned, researchers often custom-build ergometers using a motorized chain drive system (Elmer and Martin, 2013; Lewis et al., 2018). For example, Elmer and Martin (2013) described the construction of an isokinetic ECC cycle ergometer for research and training purposes. As in the ECC cycle ergometer described in this report, these authors used a commercially available frame and seat and an electric motor. However, a single electric motor was used to operate a chain drive system, as opposed to the dual-sided electric servo motors, with regenerative braking capacity, used in the current ergometer. Indeed, most studies have used semi-recumbent ECC cycle ergometers fitted with a single electric motor and chain drive system that rotate the cranks and pedals backward, without controlling how participants apply force during an ECC pedal cycle (Elmer and Martin, 2013; Leong et al., 2014; Paulsen et al., 2019). It may be that muscle activation patterns are completely in line with what would be regarded as purely ECC, when using chain-driven ECC cycle ergometers. However, given the previous reports of incoordination and difficulty in completing ECC cycling (Chasland et al., 2017; Peñailillo et al., 2017; Kan et al., 2019), this cannot be guaranteed; hence, our motivation to design the cycle presented here. Furthermore, that the participant in this current study “tripped” the ergometer once, late (13:47min) into the prescribed 15min of ECC cycling, suggests that incoordination and difficulty completing ECC cycling likely persists for longer than what would be reasonably hypothesized, based on familiarization times (three to five minutes) among naïve participants in previous studies (Chasland et al., 2017; Peñailillo et al., 2017; Kan et al., 2019). Reasons why the participant in this current study “tripped” the cycle in the latter stages of cycling are unclear and will be considered in a future study using a larger cohort.

Semi-recumbent ECC cycling requires a rider to resist a backward-rotating pedal, in order to generate a lengthening contraction of the knee extensor muscles (Green et al., 2018; Barreto et al., 2021). This can only occur when the pedal is an OPP force (Figure 2, OPP phase). Outside the OPP phase (Figure 2, non-OPP phase), any contraction of the knee extensors would be non-ECC (i.e., CON), as the contraction would move the lower limb in the direction of travel of the rotating pedal (i.e., knee extension *via* CON contraction of the quadriceps). As such, ECC cycle ergometers that lack the capacity to minimize the occurrence of non-ECC muscle contractions, during semi-recumbent ECC cycling, are likely limited in their controllability and, therefore, efficacy in producing purely ECC muscle contractions. In contrast, the current semi-recumbent ECC cycle ergometer has the capacity to limit muscle contractions to the OPP phase of ECC cycling and thereby ensures that participants are more likely to produce only ECC muscle contractions during ECC cycling. In the future, we would explore using biofeedback from the contracting muscles, for example the VM muscle, to trigger a cessation of the backward pedal rotation perhaps in place of the voltage generation. It will also be essential across a large cohort of participants, to quantify which erroneous muscle activation in tripped trials compared to non-tripped trials actually stopped the cycle, perhaps by correlating time of erroneous muscle “bursts” that deviate from a non-tripped pattern, to the time that the motor stopped (tripped trials).

In conclusion, we have designed and built a semi-recumbent ECC cycle ergometer, using dual-sided electric servo motors with regenerative braking capacity, that uses the kinetic energy generated when a rider applies an assistive force to the pedal, during the non-OPP phase, to “trip” and arrest the motor-driven pedals (Figure 2). With this ergometer, we suggest that the controllability of ECC muscle contractions during semi-recumbent ECC cycling may be improved and could be better used in place of other semi-recumbent ECC cycle ergometers, that operate using a chain drive system. Improved controllability of ECC muscle contractions would allow for more reliable conclusions to be drawn about the effectiveness of semi-recumbent ECC cycling in clinical and training studies. This study does not quantify the processes of familiarization that may occur given the constraints imposed by the design. However, our physiological data clearly demonstrate that activity produced in the lower limb during a N-OPP phase stops the cycle despite some activity in the dorsi and plantar flexors being recorded before 260° likely to control pedal position. Extensive testing is required to determine: (1) how lower limb ECC muscle activation compares to that previously documented using cycles that do not have such a trip mechanism, and (2) how our cycle determines familiarization to ECC cycle in a large cohort of participants. However, this cycle represents an important methodological advancement with respect to an attempt to isolate ECC muscle contractions during what is claimed to be ECC cycling exercise.

## Data Availability Statement

The raw data supporting the conclusions of this article will be made available by the authors, without undue reservation.

## Ethics Statement

The studies involving human participants were reviewed and approved by University of Wollongong, Human Ethics Committee. The patients/participants provided their written informed consent to participate in this study.

## Author Contributions

All of the authors contributed to writing the manuscript, suggesting improvements to the manuscript, reviewing the manuscript, and approving the submitted version of the manuscript.

## Funding

JW was supported by an Australian Government Research Training Program Scholarship.

## Conflict of Interest

The authors declare that the research was conducted in the absence of any commercial or financial relationships that could be construed as a potential conflict of interest.

## Publisher’s Note

All claims expressed in this article are solely those of the authors and do not necessarily represent those of their affiliated organizations, or those of the publisher, the editors and the reviewers. Any product that may be evaluated in this article, or claim that may be made by its manufacturer, is not guaranteed or endorsed by the publisher.
